# Anaerobic methanotrophic community of a 5346-m-deep vesicomyid clam colony in the Japan Trench

**DOI:** 10.1111/gbi.12078

**Published:** 2014-03-05

**Authors:** J Felden, S E Ruff, T Ertefai, F Inagaki, K-U Hinrichs, F Wenzhöfer

**Affiliations:** 1 Helmholtz-Max Planck Research Group for Deep Sea Ecology and Technology, Max Planck Institute for Marine MicrobiologyBremen, Germany; 2 Max Planck Institute for Marine Microbiology, Department of Molecular EcologyBremen, Germany; 3 MARUM Center for Marine Environmental Sciences and Department of Geosciences, University of BremenBremen, Germany; 4 Kochi Institute for Core Sample Research, Japan Agency for Marine-Earth Science and TechnologyKochi, Japan; 5 Helmholtz-Max Planck Research Group for Deep Sea Ecology and Technology, Alfred Wegener Institute for Polar and Marine ResearchBremen, Germany

## Abstract

*Vesicomyidae* clams harbor sulfide-oxidizing endosymbionts and are typical members of cold seep communities where active venting of fluids and gases takes place. We investigated the central biogeochemical processes that supported a vesicomyid clam colony as part of a locally restricted seep community in the Japan Trench at 5346 m water depth, one of the deepest seep settings studied to date. An integrated approach of biogeochemical and molecular ecological techniques was used combining *in situ* and *ex situ* measurements. In sediment of the clam colony, low sulfate reduction rates (maximum 128 nmol mL^−1^ day^−1^) were coupled to the anaerobic oxidation of methane. They were observed over a depth range of 15 cm, caused by active transport of sulfate due to bioturbation of the vesicomyid clams. A distinct separation between the seep and the surrounding seafloor was shown by steep horizontal geochemical gradients and pronounced microbial community shifts. The sediment below the clam colony was dominated by anaerobic methanotrophic archaea (ANME-2c) and sulfate-reducing *Desulfobulbaceae* (SEEP-SRB-3, SEEP-SRB-4). Aerobic methanotrophic bacteria were not detected in the sediment, and the oxidation of sulfide seemed to be carried out chemolithoautotrophically by *Sulfurovum* species. Thus, major redox processes were mediated by distinct subgroups of seep-related microorganisms that might have been selected by this specific abyssal seep environment. Fluid flow and microbial activity were low but sufficient to support the clam community over decades and to build up high biomasses. Hence, the clams and their microbial communities adapted successfully to a low-energy regime and may represent widespread chemosynthetic communities in the Japan Trench. In this regard, they contributed to the restricted deep-sea trench biodiversity as well as to the organic carbon availability, also for non-seep organisms, in such oligotrophic benthic environment of the dark deep ocean.

## Introduction

Cold seep communities establish where tectonic or gravitational forces push free gas, methane-rich pore water, and/or mud upward into sulfate-penetrated surface sediments (Boetius & Wenzhöfer, 2013). High energy availability at and near the sediment surface thereby supports enormous biomasses of chemosynthetic organisms such as siboglinid tubeworms, mytilid and vesicomyid bivalves, and giant sulfide-oxidizing bacteria (Sibuet & Olu, 1998; Levin, 2005; Grünke *et al*.,2012). These organisms are well adapted to access and use reduced compounds in seep sediments. For instance, most vesicomyid clams have a reduced gut system and thus rely almost entirely on their autotrophic sulfide-oxidizing endosymbionts for nutrient and energy supply (Childress *et al*., 1993; Goffredi & Barry, 2002, and references therein). To access the sulfide, they dig with their foot several centimeters into the sediment (Dubilier *et al.*, 2008), take the sulfide up, and transport it with their blood to the endosymbionts (Childress *et al.*, 1993). Some vesicomyid species are able to accumulate amounts of sulfide in their body that exceed ambient concentrations more than 60-fold (Childress *et al.*, 1993; Barry & Kochevar, 1998) and are thus found in habitats with a wide range of sulfide concentrations (0.6–20 mm; Barry *et al*., 1997; Decker *et al*., 2012; Pop Ristova *et al*., 2012). Bioturbation by the clams enhances the sulfate transport from the water column into the sediment, resulting in sulfate reduction (SR) at sediment depths that otherwise would be sulfate-limited (Wallmann *et al*., 1997; Levin *et al*., 2003; Treude *et al*., 2003). Hence, vesicomyid clams are able to populate seep sites of low geological activity, where sulfide is not found close to the sediment surface (Fischer *et al*., 2012).

In methane-enriched seep sediments, sulfide is a product of bacterial SR that is often coupled to the anaerobic oxidation of methane (AOM) mediated by consortia of anaerobic methanotrophic archaea (ANME) and sulfate-reducing bacteria (SRB; Boetius *et al*., 2000). High densities of these microbial consortia have been described in seep sediments of all continental margins from shallow waters to the deep sea (Knittel & Boetius, 2009, and references therein). The occurrence, distribution, and activity of the microbes involved in AOM have been intensively studied using different molecular ecological tools and biogeochemical measurements (Boetius *et al*., 2009; Knittel & Boetius, 2009). So far, there are three main ANME clades ANME-1, ANME-2, and ANME-3 (Hinrichs *et al*., 1999; Niemann *et al*., 2006b), which contain several sub-clades, such as thermophilic ANME-1 (Holler *et al*., 2011), ANME-2a-c (Orphan *et al*., 2001), and the recently described *Methanoperedenaceae* (Haroon *et al*., 2013). The involved SRB are close relatives of either *Desulfosarcina/Desulfococcus* or *Desulfobulbus* (Knittel *et al*., 2003; Schreiber *et al*., 2010; Kleindienst *et al*., 2012). The different ANME clades can be distinguished using methods based on nucleic acids (Orphan *et al*., 2001; Knittel *et al*., 2005; Pernthaler *et al*., 2008) and membrane lipids (Hinrichs *et al*., 1999; Elvert *et al.*, 2003; Rossel *et al.*, 2011).

In the last decade, the improvement in deep-sea technologies such as remotely operated vehicles or submersibles enabled the scientific community to explore seep ecosystems in detail by performing focused sampling and *in situ* measurements. These *in situ* investigations have significantly increased our knowledge of the small-scale variability of biodiversity and of biogeochemical activities within and between seep ecosystems (Jørgensen & Boetius, 2007; Boetius & Wenzhöfer, 2013, and references therein). However, only a few studies exist in water depths >4000 m because it is a technological challenge to access these remote abyssal habitats for sampling and *in situ* measurements (Boetius & Wenzhöfer, 2013). It is known from the Nankai Trough or the Japan Trench that cold seeps occur frequently even down to water depths of at least 7500 m (Kobayashi, 2002; Arakawa *et al.*, 2005, and reference therein). This tectonically active area hosts numerous seeps and the deepest known vesicomyid clam colonies at 6437 m (Sibuet *et al.*, 1988; Ogawa *et al.*, 1996; Fujikura *et al.*, 1999). Japan Trench seeps offer a unique opportunity to study microbial community structure and biogeochemical processes at abyssal seep ecosystems as most seep studies have been conducted at shallower sites (Sibuet *et al.*, 1988; Boetius & Wenzhöfer, 2013).

Although chemosynthetic clam colonies in the Japan Trench are known, detailed insights into the underlying biogeochemical processes and predominant microbial communities fueling these remote and high-biomass seep communities are sparse. Here, we combined analyses of sediment pore water chemistry, sediment–water interface exchange processes, as well as methane and sulfate turnover rate measurements with community analyses based on 16S rRNA genes and intact polar lipids (IPLs) to thoroughly investigate the biogeochemistry and microbial community. To our knowledge, this is the first and most comprehensive study on the functioning of an abyssal seep ecosystem using *in situ* activity measurements in the Japan Trench to date. Our main hypotheses were (i) the key biogeochemical processes in the sediment that fuel the spatially restricted clam colony are similar to those found at shallow seeps and (ii) the microbial community composition of this ecosystem differs from that of shallow seeps.

## Materials and Methods

### Seafloor observations and sampling

**Figure 1:**
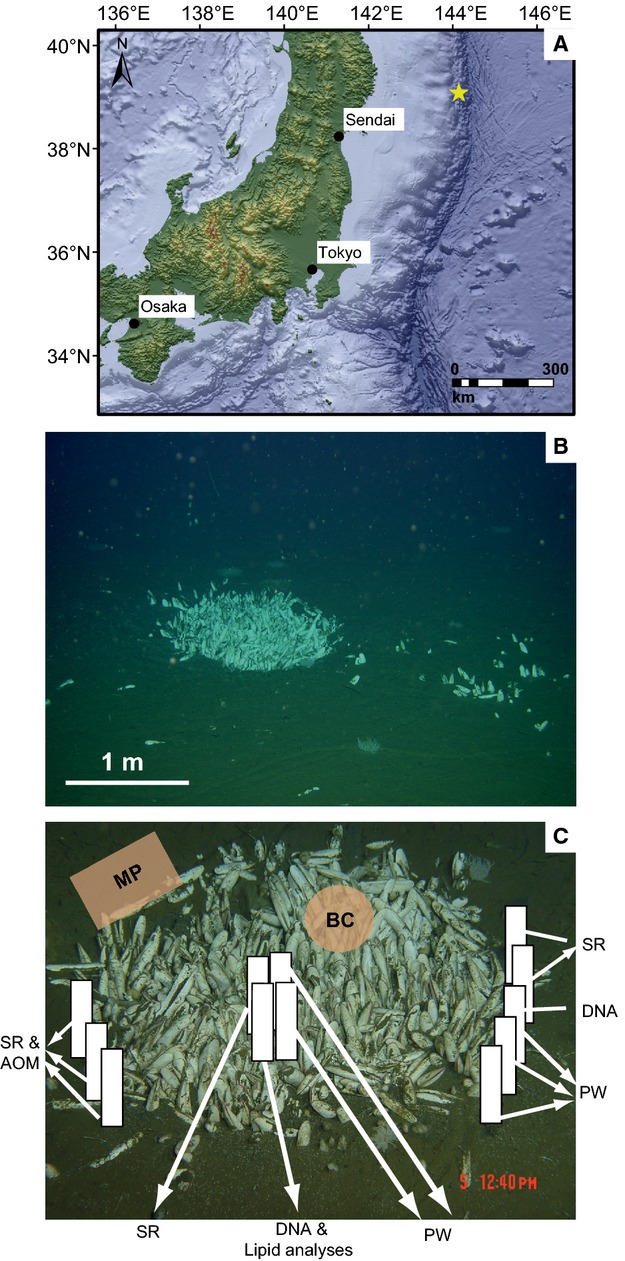
Sampling and *in situ* measurements were performed at a vesicomyid clam colony in the Japan Trench (A) at a water depth of 5346 m (A,B). The map was generated using the esri arcgis software and the General Bathymetric Chart of the Oceans (GEBCO_09 Grid, version 20091120, http://www.gebco.net). (C) Relative positions of *in situ* measurements and pushcore sampling (white bars) at the investigated clam patch. MP, microprofiler; BC, benthic chamber; DNA, 16S rDNA analyses; SR, sulfate reduction; AOM, anaerobic oxidation of methane; PW, pore water chemistry, methane concentration and isotopy; calcium carbonate, pyrite, and total organic carbon content.

During the cruise YK06-05 in 2006 with the RV *Yokosuka* to the Japan Trench, we investigated a clam colony inhabited by *Abyssogena phaseoliformis* (former known as *Calyptogena phaseoliformis*) and *Isorropodon fossajaponicum* (former known as *Calyptogena fossajaponica)* at 5346 m water depth. The names of both species were adapted according to the most recent taxonomic studies of the family *Vesicomyidae* (Krylova & Sahling, 2010, and references therein) and the accepted nomenclature in the World Register of Marine Species (http://www.marinespecies.org/). The targeted sampling and precise positioning of the *in situ* instruments were achieved with the manned research submersible *Shinkai 6500* (JAMSTEC, Nankoku, Kochi, Japan). Besides the well-defined vesicomyid clam colonies present in this area of the Japan Trench, no other chemosynthetic communities, such as sulfide-oxidizing bacterial mats, were observed. The colonies, however, were associated with different groups of benthic organisms including actiniaria, holothurians, and tube-dwelling polychaetes. Typically, the clam patches were round with diameters ranging from a few decimeters to 2 m (Fig. 1B). Distances between the widespread colonies were a few tens of meters, and we observed several trails of moving clams during the dives.

One large vesicomyid clam colony (Fig. 1A; 39°6.3560′N, 143°53.5619′E) was studied in detail with microbiological and biogeochemical methods. In the following text, this particular Japan Trench clam colony is termed JTC colony. Sampling was first performed close to the rim of the JTC colony and then at the center (Fig. 1C). Immediately after sample recovery onboard, the sediment core was sub-sampled for *ex situ* rate measurements or preserved for later analyses.

### Geochemistry

*Ex situ* pore water concentrations of sulfate and dissolved inorganic carbon (DIC) were measured, along with the concentrations and isotopic compositions of dissolved methane, total organic carbon (TOC) content, and turnover rates of sulfate as well as methane. In addition, *in situ* benthic oxygen uptake rates were determined with a microprofiler and a benthic chamber module. The data are available online via the Data Publisher for Earth & Environmental Science PANGAEA (doi: 10.1594/PANGAEA.826602).

#### Ex situ measurements

To measure the concentrations of pore water constituents, push cores were sub-sampled in 1 cm intervals and pore water was extracted via sediment squeezing (Reeburgh, 1967; 0.45 μm Durapore Filter; Millipore, Bedford, MA, USA). For each sample depth, we obtained 1–5 mL pore water that was immediately preserved and stored at 4 °C until the measurements were taken in the home laboratory. To determine sulfate concentrations, 0.5–1 mL pore water was fixed in 1 mL 2% zinc acetate (ZnAc) solution. Samples were diluted and filtered before concentrations were determined by non-suppressed anion exchange chromatography (Waters IC-Pak anion exchange column, Waters 430 conductivity detector; Waters, Milford, MA, USA). For measuring DIC concentrations, the pore water was preserved with 20 μL saturated mercuric chloride (HgCl_2_) solution and stored headspace free. DIC content of the samples was measured by the flow injection method (detector VWR scientific model 1054) according to Hall & Aller (1992). Dissolved methane concentrations and isotopic compositions were determined with the headspace method according to Kvenvolden & McDonald (1986) and Ertefai *et al*. (2010) using gas chromatography and isotope ratio mass spectrometry, respectively. Carbon isotope ratios are reported in the δ-notation as per mil (‰) deviation from Vienna Pee Dee Belemnite standard. Standard deviations of δ^13^C values were obtained from repeated measurements and were usually less than ±1.0‰.

Pyrite and carbonate content of the sediment was measured by X-ray refraction analysis as previously described (Ertefai *et al.*, 2010). TOC contents were measured from dry and homogenized sediment samples using a Leco CS 200 analyzer (LECO, St. Joseph, MI, USA). Prior to the TOC analysis, the samples were treated with 12.5% hydrogen chloride (HCl) solution to remove any inorganic carbon.

Sulfate reduction and AOM were measured *ex situ* by the whole core injection method (Jørgensen, 1978). We incubated the samples at *in situ* temperature (1.5 °C) for 48 h with either ^14^CH_4_ (dissolved in water, 2.5 kBq) or carrier-free ^35^SO_4_ (dissolved in water, 50 kBq). Sediment was fixed in 25 mL sodium hydroxide (NaOH) solution (2.5%, w/v) or 20 mL ZnAc solution (20%, w/v) for AOM or SR, respectively. Turnover rates were measured as previously described (Treude *et al.*, 2003; Kallmeyer *et al.*, 2004).

#### In situ measurements

Total oxygen uptake (TOU) and diffusive oxygen uptake (DOU) were measured at the center and the rim of the JTC colony, respectively. The difference between TOU and DOU is commonly dedicated to faunal-mediated consumption, including bioirrigation and bioturbation as well as the animal respiration itself (Glud, 2008 and references therein). TOU of the JTC colony center was determined with a small cylindrical benthic chamber module, which enclosed a sediment area of 284 cm^2^ (radius = 9.5 cm) together with 15 cm of overlying bottom water (equivalent to approximately 5 L). Two Clark-type minielectrodes continuously recorded the oxygen concentration of the enclosed water body during the incubation (Treude *et al.*, 2009). Sensors were calibrated against bottom water oxygen concentration (determined from Winkler titration) and a zero reading recorded at *in situ* temperature on board. TOU (mmol m^−2^ day^−1^) was calculated from the initial linear change in oxygen concentration vs. time (for more details see Wenzhöfer & Glud, 2002).

Oxygen penetration depth and DOU at the rim of the clam colony were measured with a small deep-sea microprofiler module (Treude *et al.*, 2009), carrying three oxygen Clark-type microelectrodes (Revsbech *et al.*, 1983) and one temperature sensor (Pt100; UST Umweltsensorentechnik GmbH, Geschwenda, Germany). High-resolution microprofiles across the sediment-water interface were measured with a vertical resolution of 100 μm on a total length of 15 cm. Oxygen electrodes had a linear response to the oxygen concentration in seawater and were calibrated *in situ* using constant readings in the bottom water (oxygen concentration determined by Winkler titration) and the anoxic parts of the sediment (Wenzhöfer *et al*., 2000; De Beer *et al.*, 2006). DOU (mmol m^−2^ day^−1^) was calculated from the measured microprofiles and Fick's first law of diffusion with DOU = *D*_0_ × (d*C*/d*z*), where *D*_0_ (1.26 × 10^−9^ m^−2^ s^−1^) is the molecular diffusion coefficient in water corrected for temperature and salinity (Li & Gregory, 1974), *C* (μm) is the solute concentration, and *z* (m) is the depth within the diffusive boundary layer (Rasmussen & Jørgensen, 1992).

### Microbial community analysis

#### IPL analyses

Before intact and free cell membrane constituents were analyzed by liquid and gas chromatography, freeze dried sediment was spiked with internal standards and lipids were extracted using a modified Bligh and Dyer method (Sturt *et al.*, 2004). The total lipid extract was separated chromatographically on a glass column using 3 g of silica gel (60 mesh) into three fractions: a non-polar fraction (dichloromethane), a glycolipid fraction (acetone), and a phospholipid fraction (methanol). The phospholipid fractions were analyzed for IPLs, which were analyzed by high performance liquid chromatography/electrospray ionization-multiple stage-mass spectrometry (HPLC/ESI-MS^n^) as previously described (Sturt *et al.*, 2004). The non-polar fractions were further separated for gas chromatography analyses, following standard protocols for separation, derivatization, and transesterification (Elvert *et al.*, 2000, 2003) described in detail by Ertefai *et al*. (2008).

## 16S rRNA gene analyses

To analyze the microbial community composition, we constructed archaeal and bacterial 16S rRNA gene libraries of sediments from the center and the rim of the JTC colony. On board, sediment cores were sectioned into 1–5 cm intervals and frozen at −20 °C. Total community DNA was retrieved from 5 g of sediment (pooled from the 0 to 10 cm depth horizon) by chloroform extraction as described by Zhou *et al*. (1996) and purified using the Wizard DNA clean-up system (Promega, Madison, WI, USA). PCRs for 16S rRNA gene libraries were carried out using the Master Taq polymerase (Eppendorf, Hamburg, Germany), 26–30 cycles and the bacterial primers GM3/GM4 (Muyzer *et al.*, 1995) or archaeal primers Arch20F/Uni1392R (Lane *et al.*, 1985; Massana *et al.*, 1997). Purification of PCR products, cloning reactions, and the sequencing of inserts were performed as previously described (Niemann *et al.*, 2006a), and chimeric sequences were removed using Mallard (Ashelford *et al.*, 2006). The 16S rRNA gene sequences were aligned with SILVA INcremental Aligner (SINA; Prüsse *et al*., 2007) and manually optimized according to the secondary structure. Phylogenetic classification was carried out using the arb software package (Ludwig *et al.*, 2004) based on the SILVA small subunit 16S rRNA reference sequence database (ssuref v111; Quast *et al.*, 2013). Phylogenetic trees were calculated with the maximum likelihood algorithm phyml (100 bootstraps) and a positional variability filter as described before (Ruff *et al.*, 2013). Operational taxonomic units at 98% 16S rRNA gene identity (OTU_0.02_) and Chao1 richness estimates were calculated using the software mothur v1.24 (Schloss *et al.*, 2009). The nucleotide sequences reported in this paper have been archived in the EMBL, GenBank, and DDBJ nucleotide sequence databases under the accession numbers HG425384–HG425704.

## Results

### Sediment solid phase

**Figure 2:**
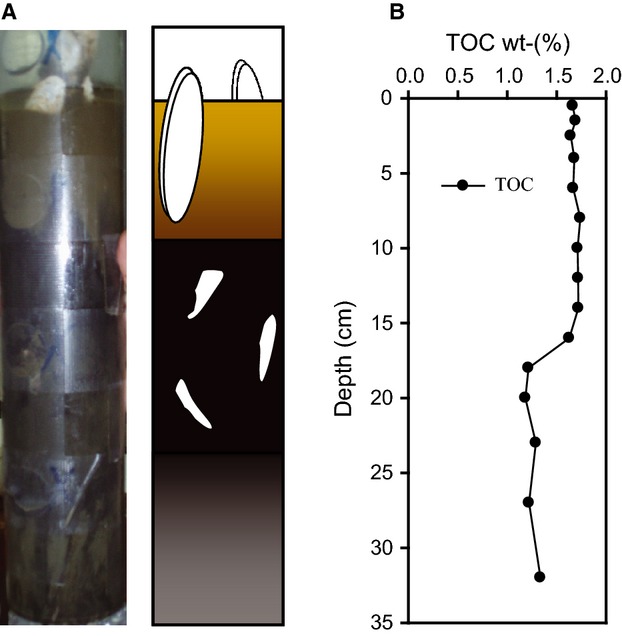
Stratigraphy and mineralogy of the sediment sampled in the center of the JTC colony. Core image and sketch (A) and total organic carbon = TOC (B).

The recovered sediment cores were visually differentiated into upper (0–10 cm below seafloor – cmbsf), middle (10–25 cmbsf), and lower (>25 cmbsf) sections (Fig. 2A). The upper 10 cm showed a light brown color and were characterized by living vesicomyid clams being partly buried into the slightly sandy sediment. The middle section of the core was black with broken shells, and a sulfidic smell was noticed during subsampling. Below 25 cm depth, the sediment was of uniform gray color. The differentiation of the sediment into different horizons was also reflected in the pyrite, carbonate, and TOC contents of the sediment (Fig. 2B). In the upper sediment horizon (0–10 cm), pyrite (FeS_2_) was absent and carbonate was low (5–7 wt-%). In the middle section, the amount of pyrite and carbonate increased to up to 8 and 32 wt-%, respectively. The carbonate content declined again in the lower section in contrast to pyrite, which reached values of up to 12 wt-%. TOC content in the sediment was constant in the upper 15 cm (approximately 1.7 wt-%), decreased in the middle section of the core (14–18 cmbsf), and remained constant again in the lower section (Fig. 2B).

### Sediment geochemistry

#### Pore water geochemistry

**Figure 3:**
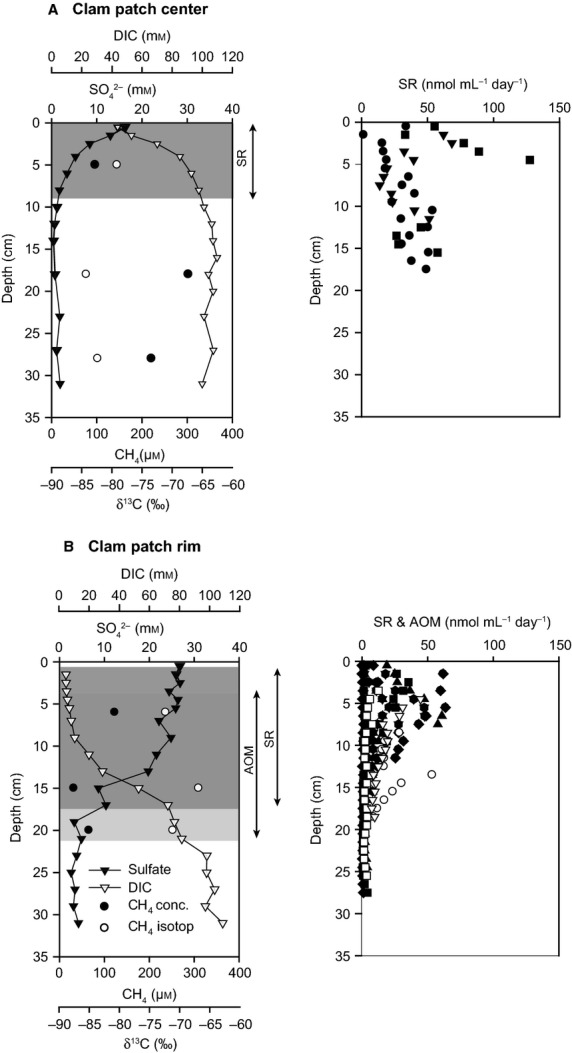
*Left panel:* Sulfate (black triangles) and DIC (white triangles) concentrations were measured in the pore water from the center (A) and the rim (B) of the JTC colony. Potential SR and AOM horizons according to pore water concentrations are highlighted. Furthermore, methane concentration (black dots) and isotopic composition (white dots) were determined at both locations. *Right panel:* SR rates (in black) and AOM rates (in white) from the center and the rim of the JTC colony. The different symbols represent replicates of turnover rate measurements.

In the center and at the rim of the JTC colony, sulfate, DIC, and dissolved methane concentrations as well as the methane isotopic composition were determined. In the center of the clam patch, sulfate concentration decreased to <1 mm at 12 cmbsf (Fig. 3A). The DIC concentration profile showed an opposite behavior to the sulfate profile, as it first increased with depth and then stayed nearly constant at more than 100 mm below 10 cmbsf. In contrast, at the JTC colony rim, sulfate penetrated deeper into the sediment (18 cmbsf) as compared to the center (Fig. 3), and the maximum DIC concentration (approximately 100 mm) was found at 31 cmbsf. Dissolved methane was analyzed in all three lithostratigraphic horizons (Fig. 3). Concentrations and isotopic compositions varied with sediment depth and differed between sampling spots. The center revealed higher dissolved methane concentrations than the rim, with a maximum between 25 and 33 cmbsf (Fig. 3A). At the center, values of dissolved methane ranged from −84 to −79‰. The highest δ^13^C values were found in the middle section of the core at 15–20 cmbsf. At the rim, the dissolved methane was less depleted in ^13^C (δ^13^C values of −72 to −66‰; Fig. 3B).

#### Methane oxidation and SR rates

Sulfate consumption was measured at the rim and the center of the JTC colony, whereas methane turnover could only be quantified in the rim sediment (Fig. 3). At the center, SR values were scattered over the investigated depth horizon and ranged from 16 to 128 nmolmL^−1^ day^−1^. The averaged depth integrated SR rate (0–16 cm) was 6.3 mmol m^−2^ day^−1^. At the colony rim, sulfate turnover was lower (1.4–64 nmol mL^−1^ day^−1^) with a maximum at about 5 cm below seafloor and decreased with increasing sediment depth. Horizontal distribution of methane consumption at the rim was similar to SR rates with values ranging from 2 to 52 nmol mL^−1^ day^−1^. The average depth (0–16 cm below seafloor)-integrated turnover rates of methane and sulfate at the rim were in the same range with 2.4 (*n* = 3) and 2.1 (*n* = 3) mmol m^−2^ day^−1^, respectively.

#### *In situ* oxygen uptake measurements

**Figure 4:**
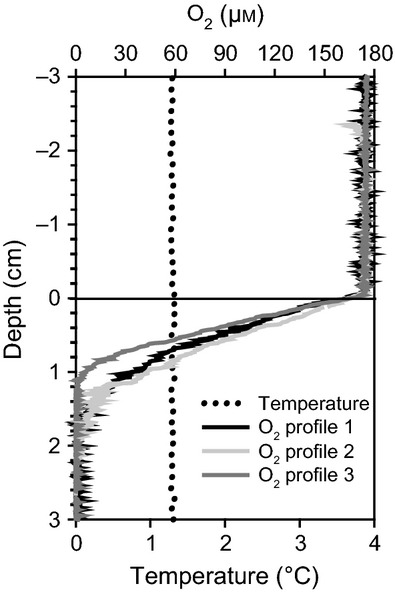
High resolution microsensor measurements outside of the colony showed an oxygen penetration depth of >1 cm and constant temperature throughout the entire sediment layer.

The microprofiler module was placed at the sediment next to the JTC colony, because a direct placement of the fragile glass sensors in the JTC colony was not possible. Approximately 20 cm beside the colony rim, the average oxygen penetration depth was 1.64 cm (*n* = 3) with an average DOU of 1.9 mmolm^−2^ day^−1^ (Fig. 4). Temperature remained constant at about 1.3 °C for the entire profiling length, and thus no heat flow was observed. In contrast to the microprofiler, the benthic chamber was placed directly on the clams, enclosing about 20 clams. A TOU of 21 mmol m^−2^ day^−1^ was measured, which is one order of magnitude higher than the DOU outside the colony. Assuming that the DOU represents the benthic oxygen consumption of the sediment in the Japan Trench at 5346 m, we calculated the oxygen consumption related to the benthic chemosynthetic community (CCOU) by subtracting DOU from TOU (CCOU = TOU − DOU). For our investigated JTC colony, this resulted in a community consumption of 19 mmol O_2_ m^−2 ^day^−1^. The chamber enclosed a sediment area of 0.0284 m^2^, populated by approximately 20 clams, which resulted in a clam density of approximately 700 clams m^−2^. Thus, one clam consumed about 27 μmol oxygen per day.

### Microbial community

#### Biomarker analyses

**Figure 5:**
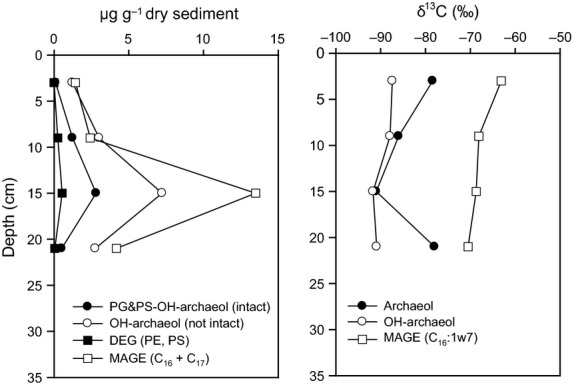
Microbial lipid profiles in the center of the JTC colony. Concentrations of archaeal and bacterial lipids are shown on the left and the isotopic compositions are on the right panel; abbreviations: PG&PS-OH-archaeol (intact): hydroxyarchaeol (OH-Ar) with phosphatidylglycerol (PG) and phosphati-dylserine (PS) as polar headgroups; OH-Ar (OH-Ar without polar headgroup); DEG (PE, PS): dialkyletherglycerolipid as phosphati-dylethanolamine and PS; MAGE (C_16_ + C_17_): sum of C_16_ and C_17_ monoalkyl glycerol ethers (MAGE), includes C_16_ and C_17_ saturated MAGE and three monounsaturated C_16_-MAGE.

Analyses of microbial lipids as both intact polar membrane lipids and free lipids were performed using sediment from below the clams in the center of the JTC colony (Fig. 5). The HPLC-MS^n^ analysis revealed phosphate-based IPLs in the form of hydroxyarchaeol (OH-Ar) with phosphatidylglycerol (PG) and phosphatidylserine (PS) as polar headgroups. Both IPL types increased with sediment depth from 0.1 to 2.8 μg g^−1^ dry sediment and were most abundant in the sediment horizon between 12 and 18 cm (2.8 μg g^−1^) before their concentration declined to 0.5 μg g^−1^ with sediment depth (Fig. 5). Bacterial dietherglycerolipids (DEG), occurring as phosphatidylethanolamine (PE) and PS, were absent in the upper 6 cm, but increased with sediment depth and peaked in the sediment horizon at 12–18 cm (0.6 μg g^−1^) and then declined to 0.1 μg g^−1^ dry sediment. The free (not intact) lipids included OH-Ar and monoalkyl glycerol ethers (MAGE). The δ^13^C values of free lipids varied with sediment depth, and the most strongly ^13^C-depleted lipids were present at 12–18 cm below seafloor (Fig. 5).

#### Phylogenetic diversity

**Figure 6:**
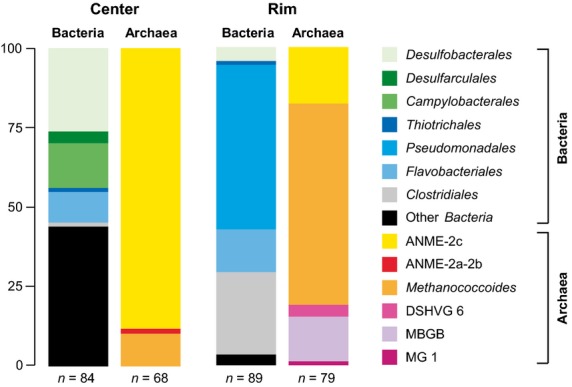
Relative 16S rRNA clone frequencies. Archaeal and bacterial diversity in the center and at the rim of the JTC colony. The scale bar represents relative clone frequencies in percent. The total number of clones per gene library is indicated below the respective column. DSHVG, Deep Sea Hydrothermal Vent Group, MBGB, Marine Benthic Group B, MG1, Marine Group 1.

Sequencing of selected clones from 16S rRNA gene libraries resulted in a total of 147 archaeal and 173 bacterial sequences (Fig. 6) from the surface sediment (0–10 cm) of the JTC colony center and the rim.

**Figure 7:**
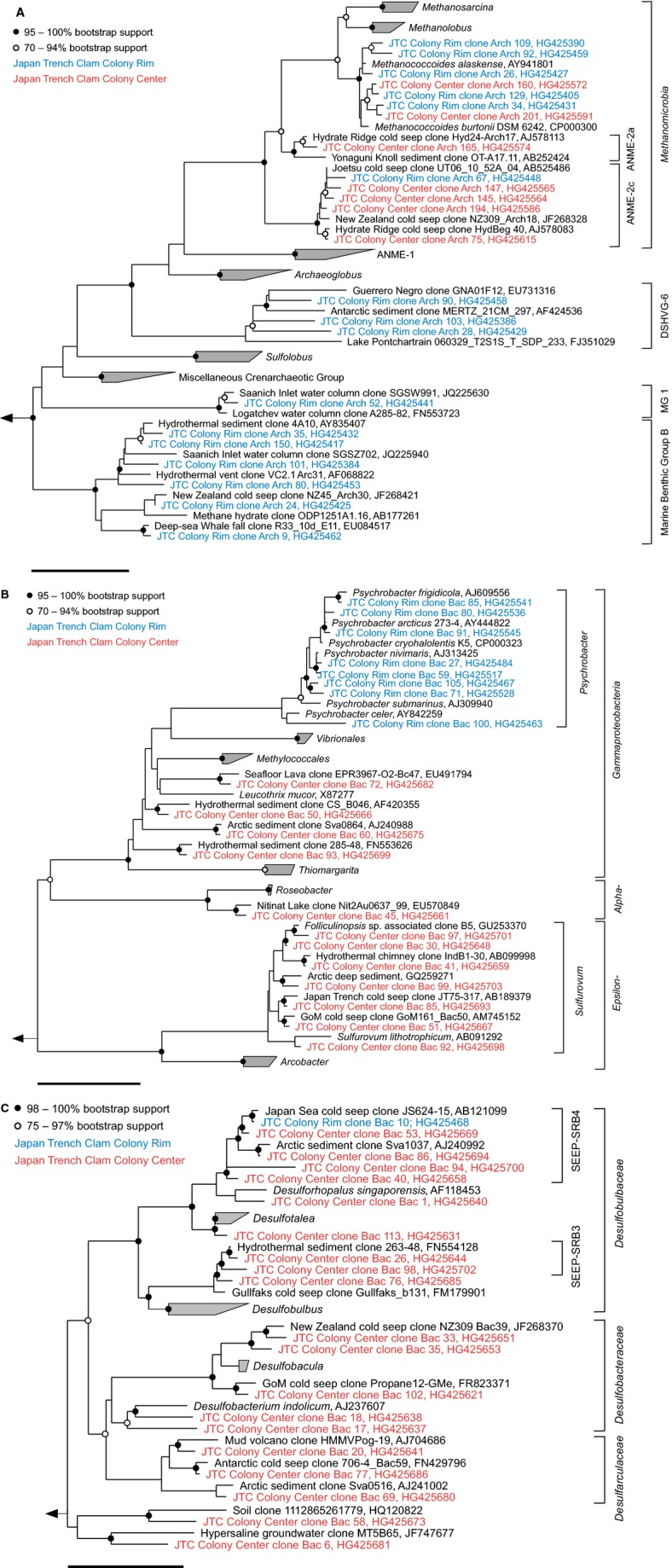
Phylogenetic affiliation of *Archaea* (A), *Alpha*-, *Gamma*-, and *Epsilonproteobacteria* (B) and *Deltaproteobacteria* (C) of the JTC colony center (red) and colony rim (blue) sediments based on 16S rRNA gene sequences. The scale bars represent 10% estimated sequence divergence.

The archaeal community of the center seemed to be extremely low in diversity (Fig. 7A), because we only obtained four OTU_0.02_, too few to reasonably estimate Chao1 richness. The OTU_0.02_ belonged to the ANME-2c clade (88% of all clones), the ANME-2a clade (1%), and the genus *Methanococcoides* (11%). The bacterial gene library of the center was more diverse (Chao1 = 41 OTU_0.02_) and was dominated by deltaproteobacterial SRB of the orders *Desulfobacterales* (26%) and *Desulfarculales* (4%). The diversity of sulfate reducers was high, including members of the genera *Desulfarculaceae*, *Desulfobacula*, *Desulforhopalus*, and *Desulfobacterium* (Fig. 7C). Interestingly, we did not detect sequences of the SEEP-SRB-1 clade, which is common at seep ecosystems, instead the SRB community seemed to be dominated by *Desulfobulbaceae* (20%), such as SEEP-SRB-3 and SEEP-SRB-4. The sulfur-oxidizing community seemed to be dominated by chemolithoautotrophic *Sulfurovum* species (14%) within the *Epsilonproteobacteria*, because sequences of *Thiotrichales* (approximately 1%) were rare. Additional clades included *Planctomycetes* and the candidate divisions JS1, OD1, and Hyd24-12 (Fig. S2).

The archaeal community of the JTC colony rim appeared to be more diverse than that of the center (Chao1 = 12 OTU_0.02_). Most sequences belonged to the genus *Methanococcoides* (63%), followed by ANME-2c archaea (18%). In addition, we found members of Marine Benthic Group B (14%), Deep-Sea Hydrothermal Vent Group 6 (4%), and Marine Group 1 (approximately 1%). The bacterial gene library of the rim sediment (Chao1 = 25 OTU_0.02_) was greatly dominated by *Psychrobacter* (51%) of the order *Pseudomonadales*, showing a high microdiversity (Fig. 7B) and also included many *Clostridiales* (Fig. S2) and several sequences of a SEEP-SRB-4 organism. The bacterial libraries of the center and rim contained distinct populations of *Flavobacteriales* and other clades (Figs S1 and S2). Remarkably, the seep sediments seemed to lack clades that are common to many seep sites worldwide, such as SEEP-SRB-1, *Methylococcales*, ANME-1, and *Thermoplasmatales*.

## Discussion

### Seepage intensity at a Japan Trench clam colony

The distribution of clams at seeps is strongly controlled by the biogeochemical processes in underlying sediments, which are influenced by the supply of methane-rich fluids from the subsurface. Upward flow of hydrocarbon-rich fluids through thrust faults (Kobayashi, 2002) at fracture zones of the Japan Trench has been described at sites, where vesicomyid clam colonies with sharp boundaries have been detected (Juniper & Sibuet, 1987; Sibuet *et al.*, 1988; Ogawa *et al.*, 1996). Oxygen and temperature profiles obtained in this study point also to a locally focused release of seep fluids at the investigated JTC colony. The measured oxygen penetration depth of 1.6 cm (Fig. 4) and the corresponding low benthic oxygen consumption rate indicated that sediments a few centimeters away from the colony rim were similar to non-seep influenced sediments (Wenzhöfer & Glud, 2002; De Beer *et al.*, 2006). At typical cold seep habitats, oxygen penetration into the sediment is usually limited to the top few millimeters (Lichtschlag *et al.*, 2010; Felden *et al.*, 2013), and is reduced even at vesicomyid clam sites that exhibit bioturbating activity (Levin *et al.*, 2003). Moreover, a straight temperature profile (Fig. 4) indicated that fluid flow from the deep subsurface was not detectable next to the colony rim. At active seep sediments, upward fluid flow is indicated by increasing temperatures with increasing depths (Feseker *et al.*, 2008). Such temperature gradients have been recorded for numerous clam colonies, for example at the Nankai Trough (Kobayashi, 2002) and at the Peruvian margin (Olu *et al.*, 1996). Unfortunately, we could not measure sediment temperature profiles directly below the clam patch. However, the concave-shaped sulfate concentration profile and the methane concentrations measured at the colony center (Fig. 3) indicated a low seepage activity nourishing the clam community. Methane δ^13^C values of <−80‰ at the JTC colony center (Fig. 3) suggested biogenic methane formation from CO_2_ and H_2_ rather than a deep subsurface thermogenic origin (Whiticar, 1999).

Alternatively, the JTC colony could have also developed, because methane-rich and sulfate-free deep sediment layers were suddenly exposed to oxygenated and sulfate-rich bottom waters as proposed for the Monterey Canyon seep ecosystems (Paull *et al.*, 2005). There, chemosynthetic benthic communities are most common on steep slopes where seafloor erosion occurs and tectonically driven fluid flow is lacking (Paull *et al.*, 2005). Erosion of the sediment in the Japan Trench might have happened when the upper sediment layer was removed during one of the regularly occurring earthquakes (Kobayashi, 2002; Kawagucci *et al.*, 2012), however, our data and dive observations do not indicate such sediment instabilities for the investigated site.

### Biogeochemical processes in sediments of the Japan Trench clam colony

To investigate whether there are also similarities to shallow seeps concerning the underlying biogeochemical processes, we analyzed methane and sulfate consumption rates. Fluid flow and associated methane availability at the JTC colony were rather low, but sufficient to maintain a dense seep community of living clams. The depth integrated rates of AOM and SR have a ratio close to one, indicating a close coupling of methane consumption and sulfide production (Treude *et al.*, 2003; Niemann *et al.*, 2006b), which constantly nourished the clams and their chemosynthetic symbionts.

Sulfate reduction rates of clam patches at different seep ecosystems cover a wide range of turnover rates (Treude *et al.*, 2003; Boetius & Suess, 2004; Pop Ristova *et al.*, 2012), and seem to correlate with the methane availability in the sediment. Rates measured at the JTC colony (maximum 64 nmol mL^−1^ day^−1^) are in the same range as those of a clam colony at the REGAB pockmarks (maximum 154 nmol mL^−1^ day^−1^; Pop Ristova *et al.*, 2012). But these values are nearly two orders of magnitude lower compared to Hydrate Ridge off Oregon, where SR rates of up to 3000 nmol mL^−1^ day^−1^ in combination with methane concentration of up to 10 mm have been found (Torres *et al.*, 2002; Boetius & Suess, 2004). In fact, even lower methane concentrations sustain clam habitats (Barry *et al.*, 1997; Wallmann *et al.*, 1997; Cambon-Bonavita *et al.*, 2009), which underlines the capability of *Vesicomyidea* clams to adapt to different environmental conditions. Low methane concentrations are not only an indicator for low seepage rates, they also result in lower sulfide availability within the sediment. *Vesicomyidea* clams are able to inhabit sites with low sulfide concentrations simply due to their ability to enrich sulfide in their body fluids above ambient concentrations (Childress *et al.*, 1993; Barry *et al.*, 1997; Barry & Kochevar, 1998). It also has been proposed that a continuous supply of sulfide is more important for these animals than the absolute concentration (Dubilier *et al.*, 2008). Furthermore, clams could influence benthic biogeochemical processes similar to vestimentiferan tubeworms, which supply the microbial community close to their roots with sulfate and thus enhance locally the microbial sulfide production (Cordes *et al.*, 2005). Furthermore, clams can move to sediments with higher sulfide concentrations as soon as sulfide is depleted at one location (Sibuet *et al.*, 1988; Olu *et al.*, 1996; Levin, 2005). In fact, such single moving clams were observed during our exploration. However, because the majority of clams were associated in patches (Fig. 1B), methane seepage and subsequent sulfide availability seemed to be sufficient to maintain the colonies.

At the JTC colony, the low methane concentrations in the surface sediments might not only be due to low seepage activity but could have also resulted from efficient methane consumption by the benthic filter. Indeed, we measured SR rates of 6.3 mmol m^−2^ day^−1^ in the sediment below the clam colony. If we assume that oxygen was used as the terminal electron acceptor for sulfide oxidation, which in turn is mainly produced by SR coupled to AOM, then TOU can be used to estimate the *in situ* methane consumption within the sediment. An oxygen uptake of 21 mmol m^−2^ day^−1^ would correspond to a methane consumption rate of 10.5 mmol m^−2^ day^−1^ based on the stoichiometric ratios of methane to sulfide (1:1) and of sulfide to oxygen (1:2). Methane efflux measurements at other clam habitats indicated that the uprising methane is completely oxidized in the sediment (Sommer *et al.*, 2006; Pop Ristova *et al.*, 2012). Therefore, the methane flux from the deep subsurface for the entire JTC colony (diameter 1.8 m^2^) would have been 19 mmol day^−1^, which corroborated that seepage was relatively low compared to other clam habitats (Torres *et al.*, 2002; Boetius & Suess, 2004; Sommer *et al.*, 2006; Pop Ristova *et al.*, 2012). This could be either a temporal effect because fluid flow may slightly vary over time at seeps (Olu *et al.*, 1996) or the seep community of the JTC colony is well adapted to efficiently use a low, but constant methane supply to build up the observed high biomasses.

### Chemosynthetic seep community at the Japan Trench clam colony

#### Clams as bioengineers

*Vesicomyid* clams rely on the biogeochemical processes in the sediment for their sulfide supply and at the same time strongly influence the benthic biogeochemical regime by bioirrigation and bioturbation (Wallmann *et al.*, 1997; Fischer *et al.*, 2012). Geochemical gradients (DIC, pyrite, calcium carbonate content) and turnover rates at the JTC colony showed an active community performing SR and AOM in the upper 10–15 cm of the sediment. However, we did not find a distinct production zone at the JTC colony, which is usually present at seep habitats of other associated organisms (Treude *et al.*, 2003; Felden *et al.*, 2010, 2013). The activity was rather spread throughout the sediment, within a depth range that was affected by the clams, which had an average body length of 15–17 cm and were buried up to four-fifths in the sediment. Clams and other seep-associated fauna, such as polychaete tubeworms, are known to enhance the availability of electron acceptors in deeper sediment horizons by bioirrigation, which results in a lowering of the sulfate methane transition zone (Wallmann *et al.*, 1997; Levin *et al.*, 2003; Treude *et al.*, 2003; Fischer *et al.*, 2012; Ruff *et al.*, 2013). By this mechanism, competing chemosynthetic surface organisms are separated from their energy source over time (Fischer *et al.*, 2012; Pop Ristova *et al.*, 2012). At the JTC colony, the clams seemed to have successfully altered the sulfide availability within the sediment and thus other common members of cold seeps such as thiotrophic bacterial mats were not observed (Treude *et al.*, 2003; Felden *et al.*, 2010, 2013; Lichtschlag *et al.*, 2010).

Using average growth rates of other vesicomyid clam species (Barry & Kochevar, 1998) and the measured shell sizes, we estimated an average age of 10–15 years for the living clams at the JTC colony. The mixture of living clams and empty shells suggested that the clam colony existed for more than 15 years; consequently, methane seepage has likely influenced this site at least for several decades. The reduced faunal diversity at the JTC colony dominated by only two clam species indicated relatively stable spatial and temporal environmental conditions (e.g., fluid flow), because it was proposed that the reduction in ecological niches and thus diversity results from habitat stability (Sibuet & Olu-Le Roy, 2002). The comparison of shallow and deep-sea seep ecosystems indicated a positive correlation between faunal diversity, biomass, and fluid flow rate independent of water depth (Sibuet & Olu-Le Roy, 2002; Cordes *et al.*, 2010). Thus, the reduced JTC colony diversity as compared to other seep ecosystems is likely due to the low fluid flow and not an effect of water depth. Contrastingly, the faunal abundance and diversity in non-seep sediments decrease with depth (Rex et al., 2006; Wei et al., 2010) as they rely on the organic carbon input from the photic zone (Smith et al., 2008). Deep cold seep communities (e.g., this study, Kobayashi, 2002; Arakawa *et al*., 2005) might therefore even influence and nourish the surrounding benthic communities at hadal depth, maintaining an active and biomass-rich benthos such as that found at continental slopes (Boetius & Wenzhöfer, 2013, and references therein).

#### Benthic microbial community in a low seepage, abyssal clam habitat

We demonstrated the presence of an active microbial community at the JTC colony that couples SR to AOM by biogeochemical measurements, lipid analyses, and 16S rRNA gene analyses. In the sediment below the clams, the lipid analyses revealed diagnostic biomarkers for AOM-specific archaeal and bacterial groups. The depth trend of IPL concentrations indicated an increase of prokaryotic cell abundances at the depth of the geochemical reaction zone, where sulfate and methane were metabolized (Fig. 5). Archaeol-based IPLs such as PI- and PG-OH-AR and high ratios of OH-Ar vs. Ar lipids strongly suggested a predominance of ANME-2 archaea in the center of the JTC colonly (Niemann & Elvert, 2008; Rossel *et al.*, 2008, 2011). The gene library results indicated that the dominant clade for anaerobic methane oxidation was ANME-2c, which is supported by previous findings (Vossmeyer *et al.*, 2012). ANME-2c seems to preferentially occur in sediments bioirrigated by clams, for example at Hydrate Ridge in the Northeast Pacific and the REGAB pockmark in the Kongo Basin (Elvert *et al.*, 2005; Knittel *et al.*, 2005; Pop Ristova *et al.*, 2012), and in low fluid flux regimes (Elvert *et al.*, 2005; Wegener *et al.*, 2008). The ANME-2c organisms that we found were closely related to those of other seeps worldwide, indicating a global distribution. Hence, the environmental niche of ANME-2c seemed to be determined by bioturbation and low methane seepage, rather than water depth and geographic location.

In contrast to other studies (Knittel *et al.*, 2003; Cambon-Bonavita *et al.*, 2009), we did not detect ANME-1 or ANME-3 in sediments below vesicomyids (Fig. 7A). The absence of ANME-1 at the JTC colony was previously observed (Vossmeyer *et al.*, 2012) and might be due to the environmental requirements of this organism. The upper sediment at the JTC colony was light brown (Fig. 2) and pyrite was absent, suggesting oxygenation due to faunal activity. ANME-1 seemed to be oxygen sensitive, because they were absent at bioirrigated seeps of Hikurangi margin (Ruff *et al.*, 2013) and increased with sediment depth and decreasing sediment irrigation at clam colonies (Knittel *et al.*, 2005; Rossel *et al.*, 2011). Additionally, ANME-1 appeared to be more sensitive to cold temperatures than ANME-2 (Rossel *et al.*, 2011). In contrast to our findings, the presence of ANME-3 in the JTC sediments was reported previously based on T-RFLP using *Hha1* (Vossmeyer *et al.*, 2012). However, ANME-3 and *Methanococcoides* spp. can not be distinguished with this method because they are closely related and include organisms that have the same restriction site for this enzyme (not shown).

The presence of sulfate-reducing *Deltaproteobacteria*, which include the partner SRB of ANME, was shown by the ^13^C-depleted IPL-derived bacterial lipids and the high amounts of MAGE (Hinrichs *et al.*, 2000; Teske *et al.*, 2002; Niemann & Elvert, 2008) in the center of the JTC colony. Remarkably, we did not detect sequences of the SEEP-SRB1 or SEEP-SRB-2 clades (Fig. 7C), which are typically the syntrophic partner SRB of ANME-2 archaea (Schreiber *et al*., 2010; Kleindienst *et al*., 2012). Instead, we found many sequences of the clades SEEP-SRB-3 and SEEP-SRB-4 within the *Desulfobulbaceae* (Fig. 7C). These clades occured as single cells in surface sediments of bacterial mat- or clam-covered seeps (Knittel *et al.*, 2003; Kleindienst *et al.*, 2012) and decreased with increasing sediment depth (Knittel *et al.*, 2003). SEEP-SRB-3 were also found at a clam colony in the Nankai trough (Li *et al.*, 1999a) and both clades occurred in bioirrigated seeps at Hikurangi margin (Ruff *et al.*, 2013). Hence, SEEP-SRB-3 and SEEP-SRB-4 might have an advantage over other SRB clades in bioturbated and thus oxygenated sediments. The occurrence of ANME-2c without their partner SRB indicated that ANME-2c organisms were either associated to other SRBs, or occurred as aggregates or single cells without direct contact to SRBs (Knittel & Boetius, 2009), or performed AOM without a partner SRB (Milucka *et al.*, 2012).

Unexpectedly, aerobic methylotrophic bacteria were not detected in the sediment of the JTC colony, although they are widespread in methane-rich ecosystems, especially in disturbed or bioirrigated cold seep sediments (Inagaki *et al.*, 2004b; Lösekann *et al*., 2007; Tavormina *et al.*, 2008; Ruff *et al.*, 2013) and contribute significantly to the benthic methane and oxygen consumption (Felden *et al.*, 2010, 2013; Boetius & Wenzhöfer, 2013). Benthic oxygen consumption at the JTC colony was in the same range as sulfide production, which also indicated that aerobic methane oxidation was low or absent. In contrast, at the REGAB clam colonies benthic oxygen consumption was up to three orders of magnitude higher than sulfide production rates (Decker *et al.*, 2012; Pop Ristova *et al.*, 2012), which was assigned to aerobic methanotrophy (Pop Ristova *et al.*, 2012).

Sulfide oxidation at the JTC colony seemed to be performed not only by the chemosynthetic vesicomyids, but also by *Sulfurovum* spp., which are sulfur oxidizers that were first isolated from hydrothermal vent sediments of the mid-Okinawa Trough (Inagaki *et al.*, 2004a). Although we cannot exclude that elemental sulfur was present in the JTC colony sediment, their occurrence indicated that *Sulfurovum* organisms are also able to oxidize sulfide (Li *et al.*, 1999a,b; Inagaki *et al.*, 2002; Fang *et al.*, 2006). *Thiotrichales* and *Arcobacter* spp., which are sulfur-oxidizing bacteria commonly detected at cold seeps (Omoregie *et al.*, 2008; Grünke *et al*., 2011, 2012), did not seem to be important at the JTC colony.

The microbial community at the rim of the JTC colony differed greatly from the one at the center, despite a distance of only 30 cm and mirrored the sharp biogeochemical gradients and defined ecosystem boundaries. The sediment at the rim of the colony was dominated by psychrophilic *Gammaproteobacteria* and comprised clades that are common to deep-sea sediments, such as *Thaumarchaeota* (Durbin & Teske, 2011). However, sequ-ences of the Marine Benthic Group B, *Desulfobacterales,* ANME-2c, and *Methanococcoides*, which occur in methane-rich subsurface sediments (Biddle *et al.*, 2006; Inagaki *et al.*, 2006), were also found indicating that methane was at least occasionally present, Nevertheless, there was little community overlap between the sediments on species-level (98% 16S rRNA gene identity; Fig. 7A–C; Figs S1 and S2), corroborating distinct differences between these seafloor habitats.

## Conclusion

In contrast to non-seep systems, a correlation of biodiversity and biomass with water depth was, so far, not found for methane seeps. However, piezophilic adaptations of the methane-oxidizing microbial community remain speculative, as only a few studies have been conducted below 5000 m water depth. Our investigation includes the first in-depth analysis of the microbial community structure and activity at an abyssal seep site. We could show that an abyssal clam colony in the Japan Trench was similar to the ones found at shallower depths, concerning the predominant biogeochemical processes, such as AOM, SR, and benthic oxygen consumption. The tight coupling of AOM and SR rates indicated that abyssal benthic methane filters are as efficient as those of shallow seeps. Our findings suggested that the environmental niche of the dominant ANME archaea and sulfate reducers may be determined by bioturbation of the clams and low methane seepage rather than by pressure or geographic location. However, other key functional populations, such as thiotrophs, differed from those found at shallow clam seeps, or seemed to be absent, indicating environmental filtering due to the extreme environment or dispersal limitation. We show that advances in deep-sea technology (Boetius & Wenzhöfer, 2013 and references therein) finally enable us to improve our limited knowledge about the biogeochemistry and microbiology of abyssal methane seeps, which occur frequently along deep-sea trenches and faults with active fluid flow (Sibuet & Olu, 1998; Tyler *et al.*, 2002; Judd, 2003) and could be significant for the marine methane budget.

## Supporting Information

Fig S1 Phylogenetic affiliation of Bacteroidetes in JTC colony center (red) and colony rim (blue) sediments based on 16S rRNA gene sequences. The scale bar represents 10% estimated sequence divergence.

Fig S2 Phylogenetic affiliation of all other bacterial sequences retrieved from JTC colony center (red) and colony rim (blue) sediments based on 16S rRNA gene sequences. The scale bar represents 10% estimated sequence divergence.
